# High CD3+ and CD34+ peripheral blood stem cell grafts content is associated with increased risk of graft-versus-host disease without beneficial effect on disease control after reduced-intensity conditioning allogeneic transplantation from matched unrelated donors for acute myeloid leukemia — an analysis from the Acute Leukemia Working Party of the European Society for Blood and Marrow Transplantation

**DOI:** 10.18632/oncotarget.8463

**Published:** 2016-03-29

**Authors:** Tomasz Czerw, Myriam Labopin, Christoph Schmid, Jan J. Cornelissen, Patrice Chevallier, Didier Blaise, Jürgen Kuball, Stephane Vigouroux, Frédéric Garban, Bruno Lioure, Nathalie Fegueux, Laurence Clement, Anna Sandstedt, Johan Maertens, Gaëlle Guillerm, Dominique Bordessoule, Mohamad Mohty, Arnon Nagler

**Affiliations:** ^1^ Department of Bone Marrow Transplantation and Oncohematology, Maria Sklodowska-Curie Memorial Cancer Centre and Institute of Oncology, Gliwice Branch, Gliwice, Poland; ^2^ Clinical Hematology and Cellular Therapy Department, The Acute Leukemia Working Party of the EBMT office, Hopital Saint-Antoine APHP Paris, France; ^3^ INSERM UMRs 938, Paris, France; ^4^ Université Pierre et Marie Curie (UPMC, Paris VI), Paris, France; ^5^ Klinikum Augsburg, University of Munich, Munich, Germany; ^6^ Department of Hematology, Erasmus University medical center Cancer Institute, Rotterdam, The Netherlands; ^7^ CHU Nantes, Dept. D'Hematologie, Nantes, France; ^8^ Unité de transplantation et de thérapie cellulaire, Institut Paoli Calmettes, Marseille, France; ^9^ University Medical Centre, Dept. of Haematology, Utrecht, The Netherlands; ^10^ CHU Bordeaux, Hôpital Haut-leveque, Pessac, France; ^11^ Hopital A. Michallon, Hématologie Clinique, Pole Cancérologie, Grenoble, France; ^12^ Nouvel Hopital Civil, Strasbourg, France; ^13^ CHU Lapeyronie, Département d'Hématologie Clinique, Montpellier, France; ^14^ Hôpital de Brabois, Centre Hospitalier Universitaire (CHU) de Nancy, Vandoeuvres les Nancy, France; ^15^ University Hospital, Dept. of Hematology, Linköping, Sweden; ^16^ University Hospital Gasthuisberg, Dept. of Hematology, Leuven, Belgium; ^17^ CHU Morvan, Brest, France; ^18^ CHRU Limoges, Service d'Hématologie Clinique, Limoges, France; ^19^ Hematology and Bone Marrow Transplantation, Chaim Sheba Medical Center, Tel Hashomer, Israel

**Keywords:** allogenic transplantation, stem cell transplantation, acute myeloid leukemia (AML), reduced-intensity conditioning, cell dose

## Abstract

Inconsistent results have been reported regarding the influence of graft composition on the incidence of graft versus host disease (GVHD), disease control and survival after reduced-intensity conditioning (RIC) allogeneic peripheral blood stem cell transplantation (allo-PBSCT). These discrepancies may be at least in part explained by the differences in disease categories, disease status at transplant, donor type and conditioning. The current retrospective EBMT registry study aimed to analyze the impact of CD3+ and CD34+ cells dose on the outcome of RIC allo-PBSCT in patients with acute myelogenous leukemia (AML) in first complete remission, allografted from HLA-matched unrelated donors (10 of 10 match). We included 203 adults. In univariate analysis, patients transplanted with the highest CD3+ and CD34+ doses (above the third quartile cut-off point values, >347 × 10^6/kg and >8.25 × 10^6 /kg, respectively) had an increased incidence of grade III-IV acute (a) GVHD (20% vs. 6%, *P* = .003 and 18% vs. 7%, *P* = .02, respectively). There was no association between cellular composition of grafts and transplant-related mortality, AML relapse, incidence of chronic GVHD and survival. Neither engraftment itself nor the kinetics of engraftment were affected by the cell dose. In multivariate analysis, CD3+ and CD34+ doses were the only adverse predicting factors for grade III-IV aGVHD (HR = 3.6; 95%CI: 1.45-9.96, *P* = .006 and 2.65 (1.07-6.57), *P* = .04, respectively). These results suggest that careful assessing the CD3+ and CD34+ graft content and tailoring the cell dose infused may help in reducing severe acute GVHD risk without negative impact on the other transplantation outcomes.

## INTRODUCTION

Reduced intensity conditioning (RIC) regimens have been developed with aim to extend the use of allogeneic hematopoietic stem cell therapy (allo-SCT) to older patients and those with pre-existing comorbidities [1]. According to data from Center for International Blood and Marrow Transplant Research (CIBMTR) and European Society for Blood and Marrow Transplantation (EBMT), the number of such procedures is steadily increasing, reaching currently 40% of all allo-SCT [2,3]. Nowadays, peripheral blood (PB) is the predominant graft source for adult allogeneic donor transplants (almost 80%) [2–4]. Compared to myeloablative (MAC) setting, in the RIC procedures the curative potential relies principally on the immunological effects of the graft against the leukemia the so called GVL effect rather than on the power of chemo- or radiotherapy [1]. Thus, cellular composition of grafts is believed to be a significant determinant of outcome. Nevertheless, the impact of PB stem cell grafts cellularity on the outcome of RIC allo-SCT is still controversial. This is mainly due to the fact that inconsistent results have been reported regarding the influence of CD3+ and CD34+ cell doses on the incidence of graft-*versus*-host disease (GVHD), disease control and survival in the studies published so far [5–13]. These discrepancies may be at least in part explained by the differences in disease categories, disease status at transplant, donor type and the degree of HLA match between donor and recipient. Furthermore, the role of CD3+ cell counts is less well characterized than that of CD34+ and was previously a part of only one report regarding unrelated donor non-myeloablative allo-SCT [13]. In the current retrospective EBMT registry study we aimed to analyze the impact of CD3+ and CD34+ cell doses in PBSC grafts on the outcome of RIC allo-SCT in a homogeneous population of patients, taking into account aforementioned factors. We focused on the population of patients with AML allografted in first complete remission (CR1) from fully matched (10/10 match) unrelated donors (MUD) after year 2000.

## RESULTS

Two hundred and three patients were enrolled. Their median age was 58 (range, 21-73). Detailed patient and transplant characteristics are summarized in (Table 1).

**Table 1:** Patient characteristics

N	203
Patient sex	
Male	116 (57%)
Female	87 (43%)
Median patient age, years (range)	58 (21-73)
AML cytogenetic risk	
Favorable	4 (2%)
Intermediate	142 (70%)
Adverse	42 (21%)
Failed	15 (7%)
Number of induction courses to achieve CR1	
1	133 (66%)
>1	70 (34%)
Interval from diagnosis to achieve CR1, days (range)	51 (21-350)
Interval from CR1 to transplantation, days (range)	115 (15-351)
Median donor age, years (range)	34 (19-61)
Donor sex	
Male	135 (67%)
Female	68 (33%)
Female donor to male recipient	32 (16%)
Patient CMV serosatus	
Negative	94 (46%)
Positive	109 (54%)
Donor CMV serostatus	
Negative	134 (66%)
Positive	69 (34%)
Conditioning	
Chemotherapy-based	143 (70%)
Fludarabine + busulfan	110
Fludarabine + treosulfan	14
Fludarabine + melphalan	12
Other	7
TBI-based	60 (30%)
In-vivo T-cell depletion	166 (82%)
ATG	153
Campath	13
GVHD prophylaxis	
CsA + Mtx	129 (64%)
CsA + MMF	69 (34%)
Other	5 (2%)
Median CD34(+) dose, cells per kilogram x 10^6 (range)	6.53 (1.34-41.3)
Interquartile range	5.0-8.25
Median CD3(+) dose, cells per kilogram x 10^6 (range)	250 (50-885)
Interquartile range	175.4-347
Median year of transplantation, range	2011 (2000-2012)
Median follow-up, months (range)	22 (3-105)

Abbreviations: AML – acute myeloid leukemia; CR1 – first complete remission; CMV – cytomegalovirus; TBI - total body irradiation; ATG - antithymocyte globulin; GVHD – graft-versus-host disease; CsA – cyclospirin A; Mtx – methotrexate; MMF - mycophenolate mofetil

The probabilities of OS and LFS at 2 years from transplantation for the whole study group were 64% (95% CI : 56-71) and 60% (53-68), respectively. The relapse rate was 24% (18-30), whereas NRM 16% (11-22). The incidence of grade II-IV acute GVHD was 31% (25-38), whereas severe grade III-IV was 10% (6-14). Chronic GVHD rate was estimated at 44% (37-52).

### Impact of graft content on the incidence of GVHD

**Figure 1:**
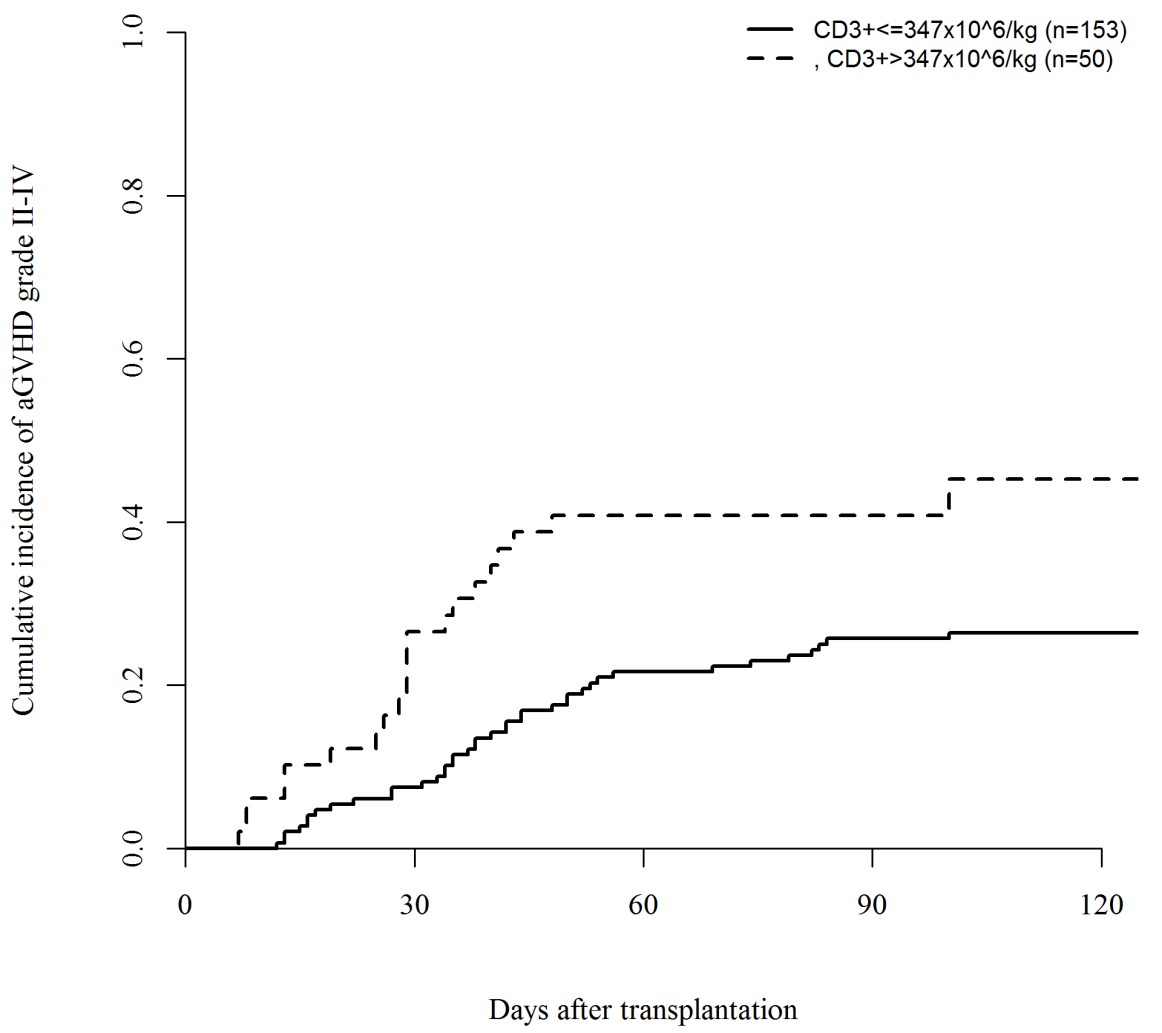
Impact of CD3+ dose on the incidence of acute GVHD grade II-IV (*P* = .007)

**Figure 2:**
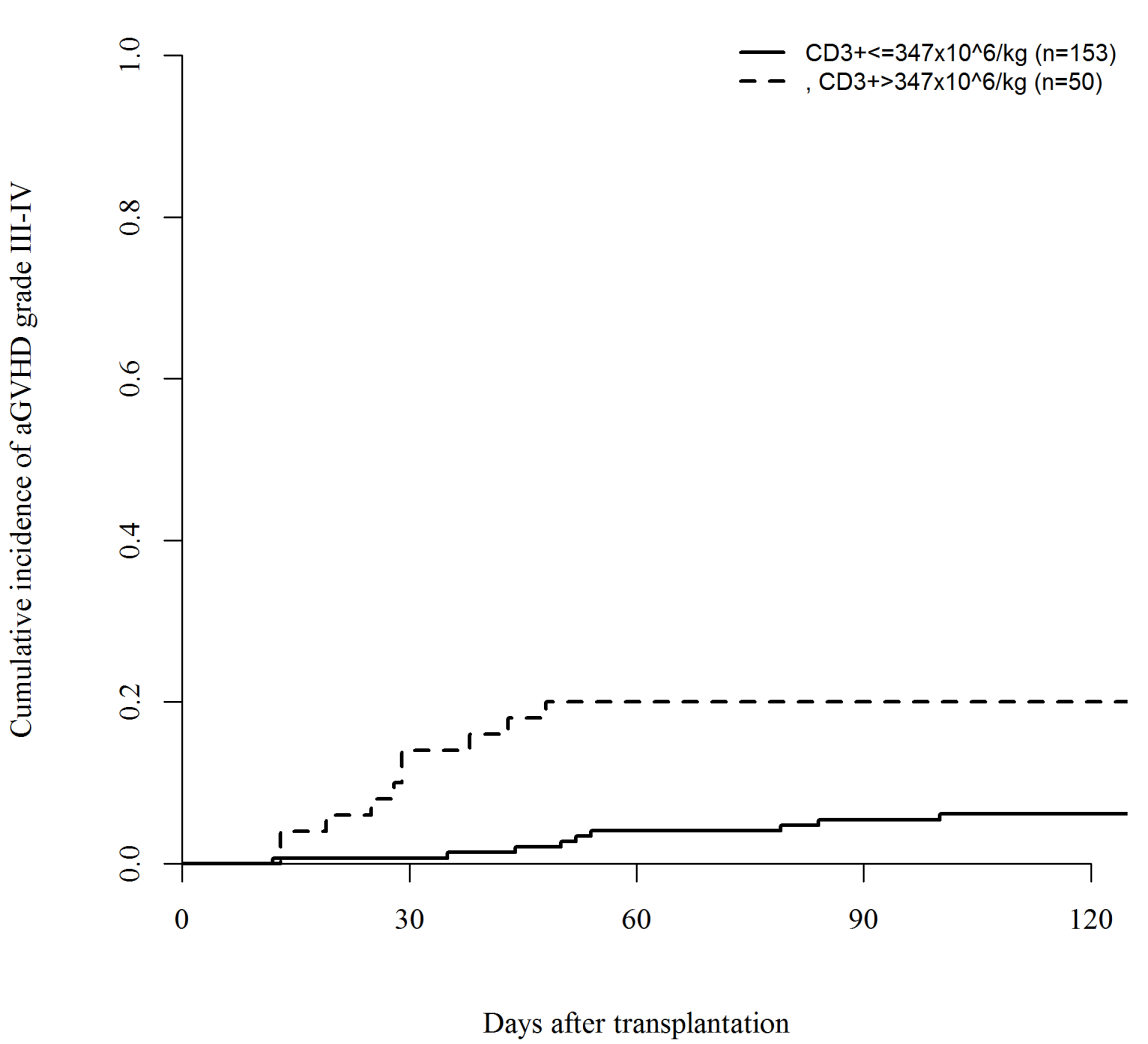
Impact of CD3+ dose on the incidence of acute GVHD grade III-IV (*P* = .003)

**Figure 3:**
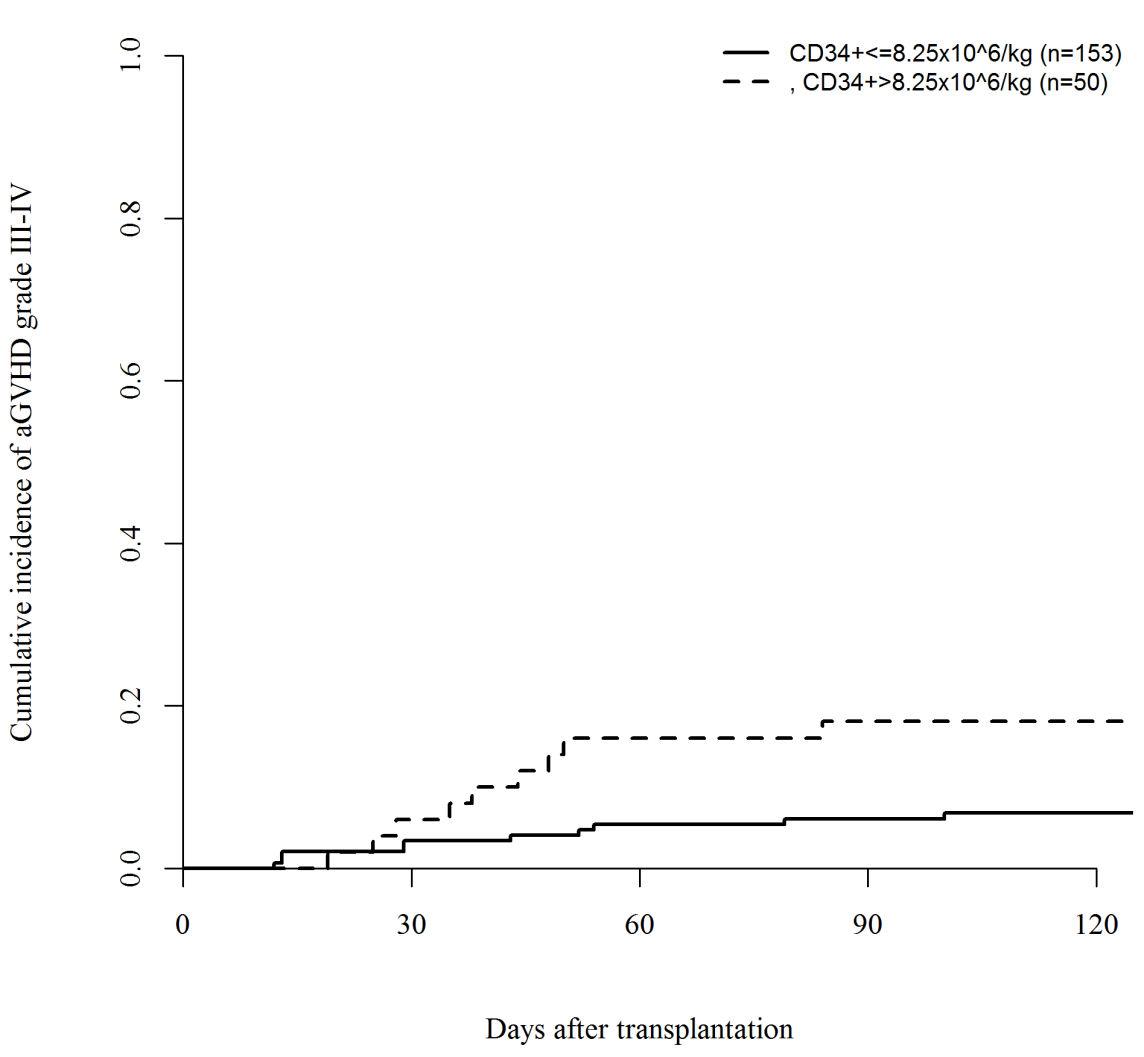
Impact of CD34+ dose on the incidence of acute GVHD grade III-IV (*P* = .02)

In a univariate analysis, patients transplanted with the highest CD3+ doses (above the third quartile cut-off point value, >347 × 10^6/kg) had an increased incidence of acute GVHD grade II-IV (45% *vs*. 26%, *P* = .007) and grade III-IV (20% *vs*. 6%, *P* = .003), respectively (Figures 1 and 2). In the quartile of patients transplanted with the highest CD34+ graft content (>8.25 × 10^6 /kg) increased incidence of grade III-IV acute GVHD (18% *vs*. 7%, *P* = .02) was observed (Figure 3). Other risk factors for acute GVHD were transplantation from CMV seropositive donors (grade II-IV; 44% *vs*. 24%, *P* = .005) and from females to males (grade III-IV; 19% *vs*. 8%, *P* = .04). There was no association between cellular composition of grafts and the incidence of chronic GVHD. Use of in-vivo T-cell depletion was a protective factor for the presence of chronic GVHD (40% *vs*. 64%, *P* = .01).

In a multivariate analysis, CD3+ dose was the only adverse predicting factor for acute GVHD grade II-IV (HR= 2.1; 95%CI: 1.25-3.55, *P* = .005) and together with CD34+ dose for acute GVHD grade III-IV (CD3+, HR=3.6; 95%CI: 1.45-9.96, *P* = .006; CD34+, HR=2.65; 95%CI: 1.07-6.57, *P* = .04). Use of in-vivo T-cell depletion was an independent predictor for lower incidence of chronic GVHD (HR= 0.43; 95%CI: 0.25-0.73, *P* = .002). There was also no significant correlation between the number of CD3+ and CD34+ cells infused (Spearman correlation coefficient: *P* = .2). However, there was a trend towards more patients transplanted with the highest CD3+ cell numbers (>347 × 10^6/kg) in in the group of patients with CD34+ cell dose higher than 8.25 × 10^6 /kg, *P* = .08.

### Impact of graft content on the engraftment

The engraftment rate equaled 99% and was not affected by CD3+ nor by the CD34+ counts. Only one patient did not engraft and one patient experienced a late graft failure. Kinetics of engraftment was not faster in patients given upper quartile CD3+ cell counts (17 days (1-18) *vs*. 17 (16-28), *P* = .93) nor in those that were given upper quartile CD34+ cell counts (18 (1-27) *vs*. 17 (6-28), *P* = .46). Likewise, the rates of full donor chimerism were not statistically higher in case of infusion of the highest CD3+ (86.8% *vs*. 79%, *P* = .28) nor CD34+ (84.2% *vs*. 79.8%, *P* = .55) cell doses.

### Impact of graft content on the other transplantation outcome

There was no association between cellular composition of grafts and AML relapse, NRM and survival (both OS and LFS). Factors that independently affected unfavorable outcome were: time from diagnosis to CR1 above median for NRM, time from CR1 to allo-SCT below median and unfavorable karyotype for LFS and OS, and unfavorable karyotype for relapse incidence. Detailed results of univariate and multivariate analyses for significant factors are presented in Tables 2 and 3, respectively.

**Table 2:** Results of univariate analysis of factors affecting outcome

		RI % at 2 years (95% CI)	NRM % at 2 years (95% CI)	LFS % at 2 years (95% CI)	OS % at 2 years (95% CI)	aGVHD II-IV % at 100 days (95% CI)	aGVHD III-IV % at 100 days (95% CI)	cGVHD % at 2 years (95% CI)
**CD34(+) dose – quartiles** (cells per kg x 10^6)	1 (1.34-5.0)	21 (10-36)	19 (8-32)	60 (45-76)	64 (49-79)	30 (18-42)	6 (2-15)	48 (32-62)
	2 (5.0-6.53)	22 (11-35)	16 (6-29)	62 (48-77)	68 (54-82)	36 (23-50)	9 (3-19)	42 (27-56)
	3 (6.53-8.25)	27 (14-42)	11 (4-22)	62 (47-78)	68 (53-83)	29 (17-42)	6 (2-15)	38 (22-53)
	4 (8.25-41.3)	26 (14-39)	19 (9-33)	54 (40-69)	55 (40-70)	30 (18-43)	18 (9-30)	46 (31-60)
	*P*-value	0.87	0.45	0.67	0.49	0.79	0.12	0.68
**CD34(+) dose < >3 quartile cut-off point value** (cells per kg x 10^6)	<8.25	23 (16-31)	15 (9-22)	62 (53-71)	67 (58-75)	31 (24-39)	7 (4-12)	43 (34-51)
	>8.25	26 (14-39)	19 (13-27)	54 (40-69)	55 (40-70)	30 (18-43)	18 (9-30)	46 (31-60)
	*P*-value	0.69	0.44	0.24	0.16	0.84	**0.02**	0.79
**CD3(+) dose – quartiles** (cells per kg x 10^6)	1 (50-175.4)	20 (10-33)	16 (7-29)	64 (49-78)	70 (56-84)	25 (14-38)	6 (2-15)	40 (25-55)
	2 (175.4-250)	28 (15-43)	16 (7-29)	56 (41-71)	65 (50-79)	31 (18-44)	6 (2-15)	52 (36-66)
	3 (250-347)	32 (18-46)	12 (5-24)	55 (40-71)	57 (41-72)	23 (12-36)	6 (2-16)	41 (26-55)
	4 (347-885)	15 (6-26)	21 (10-35)	65 (50-79)	64 (49-79)	45 (31-59)	20 (10-32)	43 (28-58)
	*P*-value	0.27	0.67	0.73	0.60	**0.04**	**0.03**	0.57
**CD3(+) dose < >3 quartile cut-off point value** (cells per kg x 10^6)	<347	27 (19-35)	15 (9-21)	59 (50-67)	64 (55-72)	26 (20-34)	6 (3-11)	44 (35-53)
	>347	15 (7-26)	21 (14-28)	65 (50-79)	64 (49-79)	45 (31-59)	20 (10-32)	43 (28-58)
	*P*-value	0.13	0.39	0.53	0.73	**0.007**	**0.003**	0.88
**Interval from diagnosis to achieve CR1**	<median	22 (14-32)	10 (5-18)	67 (57-77)	68 (58-78)	29 (21-39)	8 (4-15)	46 (34-56)
	>median	23 (15-33)	23 (15-33)	53 (42-64)	60 (49-71)	33 (24-43)	11 (6-18)	45 (34-55)
	*P*-value	0.93	**0.03**	0.13	0.23	0.59	0.49	0.86
**Interval from CR1 to transplantation**	<median	27 (18-37)	23 (14-33)	50 (39-62)	52 (41-63)	32 (23-41)	8 (4-15)	42 (30-52)
	>median	19 (11-28)	11 (6-19)	70 (60-80)	75 (66-85)	30 (21-40)	11 (6-18)	49 (38-59)
	*P*-value	0.12	0.08	**0.007**	**0.001**	0.86	0.54	0.12
**Female donor to male recipient**	No	23 (17-31)	15 (10-22)	61 (53-69)	65 (57-73)	30 (23-37)	8 (4-13)	44 (36-52)
	Yes	25 (10-43)	19 (13-26)	56 (37-75)	61 (43-78)	39 (22-55)	19 (8-35)	44 (26-60)
	*P*-value	0.91	0.35	0.51	0.39	0.21	**0.04**	0.81
**Cytogenetic risk**	Intermediate	18 (12-25)	16 (11-23)	65 (57-73)	69 (61-77)	30 (23-37)	11 (8-17)	45 (36-53)
	Adverse	44 (27-60)	15 (10-21)	41 (24-58)	43 (27-60)	37 (22-51)	2 (1-11)	40 (24-56)
	*P*-value	**0.003**	0.64	**0.02**	**0.004**	0.27	0.09	0.77
**In-vivo T-cell depletion**	No	24 (11-40)	25 (10-44)	51 (32-71)	49 (30-69)	38 (22-54)	6 (1-17)	64 (40-80)
	Yes	23 (17-30)	15 (4-32)	62 (54-70)	67 (59-75)	30 (23-37)	10 (6-16)	40 (32-48)
	*P*-value	0.53	0.27	0.16	**0.04**	0.38	0.39	**0.01**
**Donor CMV serostatus**	Negative	22 (15-30)	13 (7-20)	65 (56-74)	70 (61-79)	24 (17-32)	8 (4-13)	44 (35-53)
	Positive	27 (16-40)	22 (15-30)	50 (37-64)	51 (37-65)	44 (31-56)	12 (6-22)	44 (30-57)
	*P*-value	0.46	0.31	0.16	0.06	**0.005**	0.29	0.87
**TBI-based conditioning**	No	21 (14-28)	16 (10-23)	63 (54-71)	67 (59-76)	31 (24-39)	11 (6-17)	41 (32-49)
	Yes	30 (18-43)	16 (10-23)	54 (39-68)	55 (40-70)	30 (19-42)	7 (2-15)	53 (37-67)
	*P*-value	0.08	0.63	**0.04**	**0.04**	0.65	0.40	0.31

Abbreviations: RI – relapse incidence; NRM – non-relapse mortality; LFS - leukemia-free survival; OS-overall survival; aGVHD – acute graft-versus-host disease; cGVHD – chronic graft-versus-host disease; CI – confidence interval; CR1 – first complete remission; CMV – cytomegalovirus; TBI – total body irradiation

**Table 3:** Multivariate analyses

		*P*-value	Hazard ratio	95% CI	
				inf.	sup.
**acute GVHD II-IV**	CD3 >347 × 10^6 /kg in vivo T-cell depletion TBI	.005	2.10	1.25	3.55
		.1	.43	.15	1.18
		.16	.51	.20	1.30
**acute GVHD III-IV**	CD3 >347 × 10^6 /kg	.006	3.60	1.45	8.96
	CD34 >8.25 × 10^6 /kg	.036	2.65	1.07	6.57
	adverse cytogenetic risk	.091	.18	.02	1.32
**chronic GVHD**	in vivo T-cell depletion	.002	.43	.25	.73
**LFS**	CR1 to allo-SCT >median	.01	.54	.34	.86
	adverse cytogenetic risk	.022	1.80	1.09	2.97
**OS**	CR1 to allo-SCT >median	.003	.47	.28	.77
		.01	1.97	1.18	3.29
**RI**	CD3 >347 × 10^6 /kg	.092	.50	.22	1.12
	adverse cytogenetic risk	.001	2.76	1.49	5.13
**NRM**	Diagnosis to CR1 >median	.035	2.19	1.06	4.55

Abbreviations: GVHD – graft-versus-host disease; LFS - leukemia-free survival; OS-overall survival; RI – relapse incidence; NRM – non-relapse mortality; TBI – total body irradiation; CR1 – first complete remission; allo-SCT – allogeneic stem cell transplantation; CI – confidence interval

## DISCUSSION

In the current study we have addressed the issue of the potential impact of PB grafts composition on the outcome of RIC allo-SCT. Although this topic has been already studied, it still raises controversy because of the lack of unequivocal conclusions coming from the studies published so far [5–13, 19–21]. The discrepancies might result from the heterogeneity of patients included. In our analysis we have focused solely on patients with AML in CR1, which is the most common indication for allo-SCT (35%) [2–3]. As the number of procedures performed from unrelated donors exceeded those from siblings in the recent years we decided to analyze unrelated donor allo-SCT [2–3]. Furthermore, in order to exclude potential bias resulting from HLA disparities, we took into consideration only fully matched (10 of 10 match) donors. We have demonstrated that high CD3+ (>347 × 10^6/kg) and CD34+ (>8.25 × 10^6 /kg) cell dose content in the grafts is an independent prognostic factor associated with higher probability of severe acute GVHD grades II-IV and III-IV for CD3+ and grades III-IV for CD34+ cell dose, respectively. At the same time, we did not observe beneficial effect of such high cell doses on AML control, as it did not affect post transplantation relapse rate, and the other transplant related outcomes.

Existing data on the correlation between cell dose and transplantation outcome in the setting of RIC allo-PBSCT from unrelated donors are mainly concentrated on the impact of CD34+ cells on outcome. Our results are in accordance with a single center Swedish study that reported the association between transplantation of high doses (≥17 x10^6^ / kg) of CD34+ cells with higher incidence of acute GVHD grades II-IV [5]. In another single US center analysis transplantation of more than 4.2 x10^6^ CD34+ cells / kg was in turn correlated with less relapses and lower incidence of chronic GHVD [6]. The National Marrow Donor Program and CIBMTR were able to demonstrate in their registry based studies, in a rather heterogeneous group of patients, that transplantation of more CD34+ cells result in better outcome in terms of reducing transplant-related mortality and improving OS. The threshold values were 4.5 and 6 × 10^6 / kg, respectively [7–8]. Notably, the degree of HLA match between donor and recipient varied within studied cohorts. It is conceivable that these differences in study populations may explain discrepancies of obtained results with regard to relapse incidence and GVHD. In addition, in contrast to some of other studies we did not find association between cell dose and AML relapse, which may be related to the fact that only patients in CR1 were included in our study [6, 9–10]. It is reasonable to believe that the scenario may be different in patients with advanced disease stages. Indeed, it was previously shown that only patients with advanced disease status at transplantation (RIC from siblings) may benefit from infusion of high doses of CD34+ cells [9–10]. Similarly, we have not observed beneficial effect of higher CD34+ doses on NRM, LFS and OS in contrast to several previous publications [7–8, 10].

The potential impact of CD34+ cell dose on GVHD incidence, with similar to our threshold levels, was also demonstrated in the MAC allo-SCT setting from sibling donors. In the study by Przepiorka et al. transplantation of more than 8.2×10^6 CD34+/kg was associated with increased risk of acute GVHD II-IV [14]. Zaucha et al. reported that exceeding of 8 × 10^6^ CD34+/kg may lead to increased risk of chronic GVHD [15]. The same was found by Mohty and co-workers with similar CD34+ cutoff point of 8.3 x10^6^/kg [16]. Also Urbano-Ispizua and colleagues were able to prove, using CD34+ positively selected transplantations model (which may allow to exclude the influence of CD3+), that high number of infused CD34+ cells might have a detrimental effect on NRM mainly due to higher incidence of acute and chronic GVHD [17]. The practical clinical message of our findings is to suggest not to exceed 8.3 × 10^6 of CD34+ cells per kg in AML CR1 patients undergoing RIC allo-SCT from unrelated donors in order to try to avoid severe acute GVHD without impairing the other outcomes. This cell dose threshold nicely correlates with the values established in the previously published studies in patients undergoing myeloablative allo-SCT [14–17]. This cell dose threshold stands also in accordance with the RIC reports, we already discussed above, indicating better outcome with threshold values of at least 4.2-6.0 × 10^6^/kg [6–8]. It should be also noted, that in the first and only randomized trial looking at CD34 dose and outcome in an autologous setting higher stem cell dose (10-15 × 10^6^/kg *vs*. standard 4-6 × 10^6^/kg) did not yield a difference in post-transplant symptom burden or engraftment time [18].

The role of CD3+ cell counts is even less-well characterized. Baron et al. previously reported that high numbers of infused CD3+, CD4+, CD8+ and CD34+ cells in the setting of non-myeloablative (fludarabine and TBI 2 Gy) allo-SCT were associated with higher levels of day 28 donor T-cell chimerism [13]. In contrast, we did not reveal any relationship between CD3+ or CD34+ cell dose and engraftment rate, kinetics of engraftment nor the probability of achieving full donor chimerism. The disparity may be related to differences in the conditioning regimens used in the two studies, reasoning that the impact of cell dose on engraftment may be more important in non-myeloablative conditioning. Additional data regarding the possible role of T cell content in RIC allo-SCT setting come from studies which included both related and unrelated donors and were also more heterogeneous than our study group in terms of disease types and disease stages. Recent analysis by Reshef et al. indicates that higher CD8+ cell doses may be associated with lower RI and improved LFS and OS without increasing NRM and GVHD risk [19]. Cao at al. showed correlation between CD8+ graft content improvement of LFS in non-myeloablative setting [20]. Mohty et al. showed in turn that CD8+ cell dose affect development of acute GVHD [21]. We did not observe an influence of high CD3+ doses on AML relapse and survival but only its impact on the risk of severe acute GVHD, which partially correlates with the data by Mohty et al. Due to the limitations related to the retrospective nature of the study we were unable to analyze any CD3+ cell subsets on the outcome. As T cells are the major effector cells taking role in GVHD pathogenesis, it seems not surprising that the number of T-cells in the graft was found to be associated with acute GVHD risk. Indeed, removing T-cells from the graft by *ex vivo* T-cell depletion or *in vivo* T-cell depletion using ATG reduce the incidence of GVHD in a dose dependent way [22]. We have established the threshold value of >347 × 10^6 CD3+ cells/kg based on our patient population. However, we are aware that to recommend a certain CD3+ cell dose value to be infused in the grafts in general is more complex because of the presence of many confounding factors which may exist and modulate T cell responses including type of immunosuppressive regimen, ATG brand, dosage and timing and more [22–23]. In-vivo T-cell depletion, which is the common practice within European centers, was used in 82% of our transplants. In the vast majority of cases (104 patients, 68%), thymoglobulin at the dose 4-6 mg/kg was administrated. Such dose range was previously shown not to increase the incidence of relapses and decrease the incidence of acute and chronic GVHD [24–26]. Type of immunosuppressive regimen (CSA/MTX and CSA/MMF) was equally distributed between patients receiving the highest and lower doses of CD3+ and CD34+ cells. Due to limitations of retrospective analysis we were unable to check other post-transplant interventions which could also impact the outcome like for instance target cyclosporine levels used in particular centers. Only 18 patients received donor lymphocyte infusions which were equally distributed between the two groups receiving the highest and lower doses of CD3+ and CD34+ cells, respectively.

In conclusion, our results suggest that the incidence of severe acute GVHD post RIC allo-SCT, still a major cause of morbidity and mortality, is associated with the composition of the PBSC grafts, specifically it positively correlates with higher numbers of infused CD3+ and CD34+ cells. As graft composition can be manipulated, e.g. by cryopreservation of a part of stem cell product, careful assessing the CD3+ and CD34+ graft content and tailoring the cell dose infused may help in reducing the risk of severe acute GVHD risk and improving transplantation outcome. Prospective randomized trials looking at the impact of cell dose on outcomes should be conducted to avoid potential limitations coming from retrospective analyses published so far.

## MATERIALS AND METHODS

### Study design and data retrieval

Data for this study were provided and approved by the Acute Leukemia Working Party of the EBMT group registry. Audits are routinely performed to determine the accuracy of the data. Since 1990, patients provide informed consent authorizing the use of their personal information for research purposes.

### Criteria of selection

Inclusion criteria were as follows: 1) patients with *de novo* AML in CR1, 2) age ≥18 years, 3) allo-SCT performed from matched unrelated donor, 4) mandatory 10 out of 10 loci compatibility for HLA-A, -B, -C, -DRB1, and -DQB1, 5) peripheral blood as a source of stem cells, 6) reduced intensity conditioning regimen defined according to EBMT guidelines (Minimum Essential Data (MED-A) form) and published working definitions (i.e. based on busulfan at the total dose ≤ 8 mg/kg oral or intravenous equivalent (or other cytotoxic drugs) or on total body irradiation (TBI) ≤ 6 Gy) [27,28], 7) transplant period between January 2000 and December 2012, 7) known information on the transplanted cell dose (CD34+ and CD3+). Use of *in vivo* antithymocyte globulin (ATG) or Campath was allowed, whereas *ex vivo* T-cell depletion was an exclusion criterion.

### Patients and allo-PBSCT procedure

A total of 203 individuals met the selection criteria. Their median age was 58 (range, 21-73) and 57% of them were male. One hundred forty two (70%) of the patients had intermediate and 42 (21%) unfavorable AML cytogenetic features, while only four (2%) of them were categorized as having favorable cytogenetics. The karyotype risk category was unknown for 15 (7%) of the patients. Median donor age was 34 (19-61) years. The preparative regimen was based on chemotherapy in 143 (70%) of transplants whereas in 60 (30%) TBI was applied. In-vivo T-cell depletion was used in 166 (82%) of the transplants. Patients received GVHD prophylaxis mostly with cyclosporine and methotrexate (n=129, 64%) or with cyclosporine and mycophenolate mofetil (n=69, 34%). The CD34+ and CD3+ cell counts were determined by the cell processing laboratories at participating transplantation centers and reported to the EBMT registry. The transplanted doses were calculated based on actual body weight of the patient. The body mass index (BMI) and Karnofsky performance status did not statistically differ between patients transplanted with the highest *vs*. lower CD34+ (p=0.37 and p=0.2, respectively) and also CD3+ doses (p=0.1 and p=0.44, respectively). The median transplanted CD34(+) dose was 6.53 × 10^6 cells per kg (range, 1.34-41.3; interquartile range, 5.0-8.25). The median CD3(+) dose was 250 × 10^6 cells per kg (range, 50-885; interquartile range, 175.4-347). The median follow-up was 22 months (3-105). Detailed patient and transplant characteristics are summarized in (Table 1).

### Statistical analysis

Leukemia-free survival (LFS) was defined as survival with no evidence of relapse. Non-relapse mortality (NRM) was defined as probability of death while in CR. Acute and chronic GVHD were assessed and graded according to standard criteria [29–30]. Engraftment was defined as achieving granulocyte recovery in peripheral blood higher than 500 cells /μl for three consecutive days. The following patient's or graft characteristics were analyzed for their potential prognostic value in univariate analyzes: patient/donor age, sex-matching including female donor to male recipient, patient/donor CMV serology, time interval from diagnosis to CR1 and from CR1 to allo-SCT, cytogenetics risk group, use of *in vivo* T cell depletion, use of TBI in the conditioning regimen, infused dose of CD3 and CD34 cells. Continuous variables (CD34+ and CD3+ cell counts) were categorised as follows: each variable was first divided into four categories according to the quartiles. If the relative event rates (ratio of the observed number of events to the expected number of events in a category, assuming no variation across categories) in two or more adjacent categories (and the mean times-to-event) were not substantially different, these categories were grouped. If a linear trend was observed in the relative event rates, the variable was used as a continuous factor. Otherwise, the median was used as a cut-off point. Cumulative incidence functions (CIF) were used to estimate the probabilities for acute and chronic GVHD, neutrophil recovery, RI and NRM in a competing risks setting, since death and relapse are competing together [31]. Probabilities of overall survival (OS) and LFS were calculated using the Kaplan-Meier estimates [32]. Univariate analyses were performed using log-rank test for LFS and OS, Gray's test for CIF. All prognostic factors with a p-value lower than 0.10 on any of the endpoints in the univariate analysis were introduced in a multivariate analysis, then a stepwise backward procedure was used with a cut-off significance level of 0.05 for deleting factors in the model. Multivariate analyses were performed using Cox proportional-hazard model for OS and LFS, Fine-Gray model for other outcomes [33–34]. All tests were two-sided with type I error rate fixed at 0.05. Statistical analyses were performed with the SPSS 19 (SPSS Inc./IBM, Armonk, NY, USA) and R 3.1.2 (R Development Core Team, Vienna, Austria) software packages.

## References

[1] Sengsayadeth S, Savani BN, Blaise D, Malard F, Nagler A, Mohty M (2015). Reduced intensity conditioning allogeneic hematopoietic cell transplantation for adult acute myeloid leukemia in complete remission - a review from the Acute Leukemia Working Party of the EBMT. Haematologica.

[2] Pasquini MC, Zhu X Current uses and outcomes of hematopoietic stem cell transplantation: 2014 CIBMTR Summary Slides.

[3] Passweg JR, Baldomero H, Bader P, Bonini C, Cesaro S, Dreger P, Duarte RF, Dufour C, Falkenburg JH, Farge-Bancel D, Gennery A, Kröger N, Lanza F (2015). Hematopoietic SCT in Europe 2013: recent trends in the use of alternative donors showing more haploidentical donors but fewer cord blood transplants. Bone Marrow Transplant.

[4] Nagler A, Labopin M, Shimoni A, Niederwieser D, Mufti GJ, Zander AR, Arnold R, Greinix H, Cornelissen JJ, Jackson GH, Craddock C, Bunjes DW, Ganser A (2012). Mobilized peripheral blood stem cells compared with bone marrow as the stem cell source for unrelated donor allogeneic transplantation with reduced-intensity conditioning in patients with acute myeloid leukemia in complete remission: an analysis from the Acute Leukemia Working Party of the European Group for Blood and Marrow Transplantation. Biol Blood Marrow Transplant.

[5] Remberger M, Mattsson J, Hassan Z, Karlsson N, LeBlanc K, Omazic B, Okas M, Sairafi D, Ringdén O (2008). Risk factors for acute graft-*versus*-host disease grades II-IV after reduced intensity conditioning allogeneic stem cell transplantation with unrelated donors: a single centre study. Bone Marrow Transplant.

[6] Nakamura R, Auayporn N, Smith DD, Palmer J, Sun JY, Schriber J, Pullarkat V, Parker P, Rodriguez R, Stein A, Rosenthal J, Wang S, Karanas C, Gaal K, Senitzer D, Forman SJ (2008). Impact of graft cell dose on transplant outcomes following unrelated donor allogeneic peripheral blood stem cell transplantation: higher CD34+ cell doses are associated with decreased relapse rates. Biol Blood Marrow Transplant.

[7] Pulsipher MA, Chitphakdithai P, Logan BR, Leitman SF, Anderlini P, Klein JP, Horowitz MM, Miller JP, King RJ, Confer DL (2009). Donor, recipient, and transplant characteristics as risk factors after unrelated donor PBSC transplantation: beneficial effects of higher CD34+ cell dose. Blood.

[8] Törlén J, Ringdén O, Le Rademacher J, Batiwalla M, Chen J, Erkers T, Ho V, Kebriaei P, Keever-Taylor C, Kindwall-Keller T, Lazarus HM, Laughlin MJ, Lill M (2014). Low CD34 dose is associated with poor survival after reduced-intensity conditioning allogeneic transplantation for acute myeloid leukemia and myelodysplastic syndrome. Biol Blood Marrow Transplant.

[9] Perez-Simon JA, Diez-Campelo M, Martino R, Sureda A, Caballero D, Canizo C, Brunet S, Altes A, Vazquez L, Sierra J, Miguel JF (2003). Impact of CD34+ cell dose on the outcome of patients undergoing reduced-intensity-conditioning allogeneic peripheral blood stem cell transplantation. Blood.

[10] Gómez-Almaguer D, Gómez-Peña Á, Jaime-Pérez JC, Gómez-Guijosa MÁ, Cantú-Rodríguez O, Gutiérrez-Aguirre H, Martínez-Cabriales SA, García-Rodríguez F, Olguín-Ramírez LA, Salazar-Riojas R, Méndez-Ramírez N (2013). Higher doses of CD34+ progenitors are associated with improved overall survival without increasing GVHD in reduced intensity conditioning allogeneic transplant recipients with clinically advanced disease. J Clin Apher.

[11] Dhédin N, Prébet T, De Latour RP, Katsahian S, Kuentz M, Piard N, Réa D, Norol F, Jouet JP, Ribeil JA, Tabrizi R, Rio B, Lioure B (2012). Extensive chronic GVHD is associated with donor blood CD34+ cell count after G-CSF mobilization in non-myeloablative allogeneic PBSC transplantation. Bone Marrow Transplant.

[12] Martin PS, Li S, Nikiforow S, Alyea EP, Antin JH, Armand P, Cutler CS, Ho VT, Kekre N, Koreth J, Luckey CJ, Ritz J, Soiffer RJ (2016). Infused total nucleated cell dose is a better predictor of transplant outcomes than CD34+ cell number in reduced-intensity mobilized peripheral blood allogeneic hematopoietic cell transplantation. Haematologica.

[13] Baron F, Maris MB, Storer BE, Sandmaier BM, Panse JP, Chauncey TR, Sorror M, Little MT, Maloney DG, Storb R, Heimfeld S (2005). High doses of transplanted CD34+ cells are associated with rapid T-cell engraftment and lessened risk of graft rejection, but not more graft-*versus*-host disease after nonmyeloablative conditioning and unrelated hematopoietic cell transplantation. Leukemia.

[14] Przepiorka D, Smith TL, Folloder J, Khouri I, Ueno NT, Mehra R, Körbling M, Huh YO, Giralt S, Gajewski J, Donato M, Cleary K, Claxton D (1999). Risk factors for acute graft-*versus*-host disease after allogeneic blood stem cell transplantation. Blood.

[15] Zaucha JM, Gooley T, Bensinger WI, Heimfeld S, Chauncey TR, Zaucha R, Martin PJ, Flowers ME, Storek J, Georges G, Storb R, Torok-Storb B (2001). CD34 cell dose in granulocyte colony-stimulating factor-mobilized peripheral blood mononuclear cell grafts affects engraftment kinetics and development of extensive chronic graft-*versus*-host disease after human leukocyte antigen-identical sibling transplantation. Blood.

[16] Mohty M, Bilger K, Jourdan E, Kuentz M, Michallet M, Bourhis JH, Milpied N, Sutton L, Jouet JP, Attal M, Bordigoni P, Cahn JY, Sadoun A (2003). Higher doses of CD34+ peripheral blood stem cells are associated with increased mortality from chronic graft-*versus*-host disease after allogeneic HLA-identical sibling transplantation. Leukemia.

[17] Urbano-Ispizua A, Carreras E, Marín P, Rovira M, Martínez C, Fernández-Avilés F, Xicoy B, Hernández-Boluda JC, Montserrat E (2001). Allogeneic transplantation of CD34(+) selected cells from peripheral blood from human leukocyte antigen-identical siblings: detrimental effect of a high number of donor CD34(+) cells?. Blood.

[18] Shah N, Shi Q, Williams LA, Mendoza TR, Wang XS, Reuben JM, Dougherty PM, Bashir Q, Qazilbash MH, Champlin RE, Cleeland CS, Giralt SA (2016). Higher Stem Cell Dose Infusion after Intensive Chemotherapy Does Not Improve Symptom Burden in Older Patients with Multiple Myeloma and Amyloidosis. Biol Blood Marrow Transplant.

[19] Reshef R, Huffman AP, Gao A, Luskin MR, Frey NV, Gill SI, Hexner EO, Kambayashi T, Loren AW, Luger SM, Mangan JK, Nasta SD, Richman LP (2015). High Graft CD8 Cell Dose Predicts Improved Survival and Enables Better Donor Selection in Allogeneic Stem-Cell Transplantation With Reduced-Intensity Conditioning. J Clin Oncol.

[20] Cao TM, Shizuru JA, Wong RM, Sheehan K, Laport GG, Stockerl-Goldstein KE, Johnston LJ, Stuart MJ, Grumet FC, Negrin RS, Lowsky R (2005). Engraftment and survival following reduced-intensity allogeneic peripheral blood hematopoietic cell transplantation is affected by CD8+ T-cell dose. Blood.

[21] Mohty M, Bagattini S, Chabannon C, Faucher C, Bardou VJ, Bilger K, Vey N, Gaugler B, Stoppa AM, Coso D, Ladaique P, Olive D, Viens P, Blaise D (2004). CD8+ T cell dose affects development of acute graft-*vs*-host disease following reduced-intensity conditioning allogeneic peripheral blood stem cell transplantation. Exp Hematol.

[22] Bacigalupo A (2005). Antilymphocyte/thymocyte globulin for graft *versus* host disease prophylaxis: efficacy and side effects. Bone Marrow Transplant.

[23] Rubio MT, Labopin M, Blaise D, Socié G, Contreras RR, Chevallier P, Sanz MA, Vigouroux S, Huynh A, Shimoni A, Bulabois CE, Caminos N, López-Corral L (2015). The impact of graft-*versus*-host disease prophylaxis in reduced-intensity conditioning allogeneic stem cell transplant in acute myeloid leukemia: a study from the Acute Leukemia Working Party of the European Group for Blood and Marrow Transplantation. Haematologica.

[24] Baron F, Labopin M, Blaise D, Lopez-Corral L, Vigouroux S, Craddock C, Attal M, Jindra P, Goker H, Socié G, Chevallier P, Browne P, Sandstedt A (2014). Impact of *in vivo* T-cell depletion on outcome of AML patients in first CR given peripheral blood stem cells and reduced-intensity conditioning allo-SCT from a HLA-identical sibling donor: a report from the Acute Leukemia Working Party of the European Group for Blood and Marrow Transplantation. Bone Marrow Transplant.

[25] Remberger M, Ringdén O, Hägglund H, Svahn BM, Ljungman P, Uhlin M, Mattsson J (2013). A high antithymocyte globulin dose increases the risk of relapse after reduced intensity conditioning HSCT with unrelated donors. Clin Transplant.

[26] Crocchiolo R, Esterni B, Castagna L, Fürst S, El-Cheikh J, Devillier R, Granata A, Oudin C, Calmels B, Chabannon C, Bouabdallah R, Vey N, Blaise D (2013). Two days of antithymocyte globulin are associated with a reduced incidence of acute and chronic graft-*versus*-host disease in reduced-intensity conditioning transplantation for hematologic diseases. Cancer.

[27] Bacigalupo A, Ballen K, Rizzo D, Giralt S, Lazarus H, Ho V, Apperley J, Slavin S, Pasquini M, Sandmaier BM, Barrett J, Blaise D, Lowski R, Horowitz M (2009). Defining the intensity of conditioning regimens: working definitions. Biol Blood Marrow Transplant.

[28] Giralt S, Ballen K, Rizzo D, Bacigalupo A, Horowitz M, Pasquini M, Sandmaier B (2009). Reduced-intensity conditioning regimen workshop: defining the dose spectrum. Report of a workshop convened by the center for international blood and marrow transplant research. Biol Blood Marrow Transplant.

[29] Przepiorka D, Weisdorf D, Martin P, Klingemann HG, Beatty P, Hows J, Thomas ED (1995). 1994 Consensus Conference on Acute GVHD Grading. Bone Marrow Transplant.

[30] Shulman HM, Sullivan KM, Weiden PL, McDonald GB, Striker GE, Sale GE, Hackman R, Tsoi MS, Storb R, Thomas ED (1980). Chronic graft-*versus*-host syndrome in man. A long-term clinicopathologic study of 20 Seattle patients. Am J Med.

[31] Gooley TA, Leisenring W, Crowley JA, Storer BE (1999). Estimation of failure probabilities in the presence of competing risks: New representations of old estimators. Stat Med.

[32] Kaplan EL, Meier P (1958). Non parametric estimation from incomplete observations. J Am Stat Assoc.

[33] Cox DR (1972). Regression models and life tables. J R Stat Soc.

[34] Fine JP, Gray RJ (1999). A proportional hazards model for subdistribution of a competing risk. Journal of American Statistical Association.

